# *Toxoplasma gondii* Triggers Neutrophil Extracellular Traps Release in Dogs

**DOI:** 10.3389/fcimb.2020.00429

**Published:** 2020-09-25

**Authors:** Zhengkai Wei, Zedong Wang, Xiao Liu, Chaoqun Wang, Zhen Han, Di Wu, Yong Zhang, Xichen Zhang, Zhengtao Yang, Quan Liu

**Affiliations:** ^1^College of Life Sciences and Engineering, Foshan University, Foshan, China; ^2^Key Laboratory of Zoonosis, Ministry of Education, College of Veterinary Medicine, Jilin University, Changchun, China

**Keywords:** *Toxoplasma gondii*, neutrophils extracellular traps, dogs, Rac1, ROS

## Abstract

*Toxoplasma gondii* (*T. gondii*) can cause zoonotic toxoplasmosis worldwide. Neutrophil extracellular traps (NETs) have been known as a novel effector mechanism against *T. gondii* infection in the innate system of humans, cats, and sheep. Dogs are the intermediate host of *T. gondii*, in which the use of NETs against *T. gondii* infection remains unclear. Thus, this study aims to examine the effects of *T. gondii* on NETs release in dogs, and to further investigate the mechanism involved in the process. *T. gondii*-triggered NETs were analyzed by scanning electron microscopy (SEM) and fluorescence confocal microscopy, and the mechanism of *T. gondii*-triggered NETs release was determined by using inhibitors and a fluorometric reader. The results showed that *T. gondii* tachyzoites significantly triggered NETs-like structures, which consisted of DNA decorated with neutrophil elastase (NE) and myeloperoxidase (MPO). Further investigations revealed that reactive oxygen species (ROS)-, NADPH oxidase-, Rac 1- or p38 mitogen-activated protein kinase (MAPK)-signaling pathways were relevant to *T. gondii* tachyzoites-triggered NETs release. Moreover, zymosan-triggered NETs release was strikingly degraded by *T. gondii* tachyzoites treatment, indicating that *T. gondii* may escape from the NETs-based capture strategy. Taken together, promoting NETs release is suggested to limit motility and evade infection of *T. gondii* in dogs.

## Introduction

*Toxoplasma gondii* is considered as one of the most prevalent zoonotic parasites worldwide (Tenter et al., 2000; Dubey and Jones, 2008; Dubey and Dubey, 2010; Lopes et al., 2014; Pan et al., 2017). It may cause asymptomatic infection or severe symptomatic infection including developmental defects, early embryonic death, stillbirth, or abortion to *T. gondii-*infected pregnancies (Dubey and Jones, 2008; Dubey and Dubey, 2010; Pan et al., 2017). Dogs are the intermediate hosts, and multiple studies have demonstrated the prevalence of *T. gondii* in dogs in a variety of countries or areas worldwide (Jiang et al., 2015; Zhang et al., 2015; Dubey et al., 2016; Rengifo-Herrera et al., 2017; Zarra-Nezhad et al., 2017). Although these serological surveys and diagnosis analysis of dogs infected *T. gondii* have been reported, the interactions between the host dog and the parasite *T. gondii* remain not fully understood.

Neutrophil extracellular traps (NETs) are reported as a novel effector mechanism of polymorphonuclear neutrophils (PMNs) in the host immune system against infection. Since NETs were first discovered to entrap and kill extracellular bacteria (Brinkmann et al., 2004), this novel effector mechanism has also been reported to be involved in immune system response against several pathogens, such as viruses (Wardini et al., 2010; Saitoh et al., 2012), fungi (Jin et al., 2016; Urban and Nett, 2018), or parasites (Munoz-Caro et al., 2015a; Wei et al., 2016). It also has been shown that NETs against *T. gondii* infection exist in a variety of species including humans (Abi Abdallah et al., 2012), sheep (Yildiz et al., 2017), mice (Abi Abdallah et al., 2012), cattle (Yildiz et al., 2017), cats (Lacerda et al., 2019), harbor seals (Reichel et al., 2015), and dolphins (Imlau et al., 2020). NETs release can damage or kill *T. gondii*, but whether the NETs-based effector mechanism during *T. gondii* infection also works in the innate immune system of dogs remains unknown.

Various studies have demonstrated that the mechanism of NETs release is associated with multiple molecular or signaling pathways, such as NADPH oxidase, Rac, or p38 MAPK signaling pathways (Lim et al., 2011; Munoz-Caro et al., 2015a; Wei et al., 2016, 2018a; Gavillet et al., 2018). Furthermore, *T. gondii* triggered-NETs release in harbor seals is also a NADPH oxidase-, NE- and MPO-, store-operated calcium entry (SOCE)-dependent process (Reichel et al., 2015). The key molecular and signaling pathways involved in *T. gondii-*triggered NETs release in dogs still needs further investigation. In this study, we examined for the first time if NETs release in dogs during *T. gondii* infection, and also investigated the potential mechanism undergoing these processes.

## Materials and Methods

### Parasites

*Toxoplasma gondii* tachyzoites of RH strain were inoculated and cultured in Vero cells at 37°C and 5% CO_2_, *T. gondii* tachyzoites were harvested by passing through a 20 mL syringe and a 27-gauge needle three times and was purified by filtrum (5 μm). Finally, these tachyzoites were centrifuged at 3,000 r/min for 10 min and washed two times.

### Experimental Animals

Blood was collected from three healthy adult dogs, and the PMNs were isolated in the available dog PMN isolation kit® (TianJin HaoYang Biological Manufacture CO., China) as previously described (Wei et al., 2016, 2018b).

### Ethics Statement

Animal experiments were approved by the Ethics Committee on the Care and Use of Laboratory Animals of Foshan University and in accordance with the current Animal Protection Laws of China.

### Scanning Electron Microscopy (SEM) Analysis

*T. gondii* tachyzoite-triggered NETs release were examined by SEM as previously described (Wei et al., 2016). In brief, PMNs were incubated with viable *T. gondii* tachyzoites at the ratio of 1:1 for 90 min. After the specimens were fixed with 4.0% glutaraldehyde, and post-fixed with 1.0% osmium tetroxide (Merck), they were observed under scanning electron microscope (Hitachi S-3400N, Japan).

### Fluorescence Confocal Microscopy Analysis

The components of *T. gondii* tachyzoite-triggered NETs were detected with fluorescence confocal microscopy analysis as described elsewhere (Wei et al., 2016, 2018b). Briefly, the newly isolated PMNs were incubated with *T. gondii* tachyzoites at the ratio of 1:1 for 90 min. After being fixed with paraformaldehyde, the specimens were permeabilized with Triton X-100 and blocked with goat serum/PBS. Finally, the specimens were incubated with the specific antibodies against proteins in *T. gondii* tachyzoites-triggered NETs structures. In parallel experiments, PMNs were co-treated with zymosan (1 mg/mL) and *T. gondii* tachyzoites for 120 min. The NETs specific antibodies included the anti-histone antibody (LS-C353149; Life Span BioSciences, Inc) and the anti-myeloperoxidase (MPO) antibody (Orb16003; Biorbyt). DNA in *T. gondii* tachyzoites-triggered NETs structures were stained with 5 μM Sytox Orange (Invitrogen), and the images were observed and analyzed with a scanning confocal microscope (Olympus FluoView FV1000).

### Quantitation of *T. gondii*-Triggered NETs

PMNs were incubated with viable *T. gondii* tachyzoites (ratio 1:1, 1:3, or 1:6) for 90 min. To determine if *T. gondii*-triggered NETs were time-dependent, PMNs were incubated with viable *T. gondii* tachyzoites (ratio 1:1) for 10, 30, 60, 90, or 120 min. In inhibition tests, cells were pre-treated with 10 μM of the NADPH oxidase inhibitor Diphenyleneiodonium chloride (DPI, Sigma-Aldrich), 10 μM of the p38 MAPK (Sigma-Aldrich) signaling pathway inhibitor SB202190, 100 μM of the Rac1 activation inhibitor NSC23766, or 8.0 nM of the NLRP3 inhibitor MCC950 for 15 min. DNase I (90 U) was also pre-treated with cells for 15 min. Zymosan (1 mg/mL) was used as positive control. Finally, the release of *T. gondii*-triggered NETs was quantified with Pico Green® (Invitrogen) and the fluorometric reader Infiniti M200® (Tecan, Austria).

### Reactive Oxygen Species (ROS) Levels Analysis

To determine ROS levels in the process of *T. gondii*-triggered NETs release, PMNs were incubated with viable *T. gondii* tachyzoites (ratio 1:1 or 1:2) for 90 min. *T. gondii* tachyzoites-induced ROS levels in the process of NETs release were tested by using 2, 7 dichlorofluorescein diacetate (Sigma-Aldrich) and the fluorometric reader Infiniti M200® (Tecan, Austria).

### LDH Levels Analysis

To further differentiate *T. gondii* tachyzoites-triggered NETosis from necrosis, LDH levels were tested. PMNs were incubated with viable *T. gondii* tachyzoites (ratio 1:1, 1:2, or 1:4) for 90 min. *T. gondii* tachyzoites-induced LDH levels in the process of NETs release were tested by the LDH Cytotoxicity Assay kit® (Beyotime Biotechnology, China).

### Statistical Analysis

Data were expressed as the means ± standard deviation (SD). One-way analysis of variance (ANOVA) with Tukey's multiple comparison tests was performed by using the GraphPad 5.0 software to analyze the differences among groups. The level of *P* ≤ 0.05 was considered as significant.

## Results

### *T. gondii* Tachyzoites Triggered Dog NETs Release

**Figure 1:**
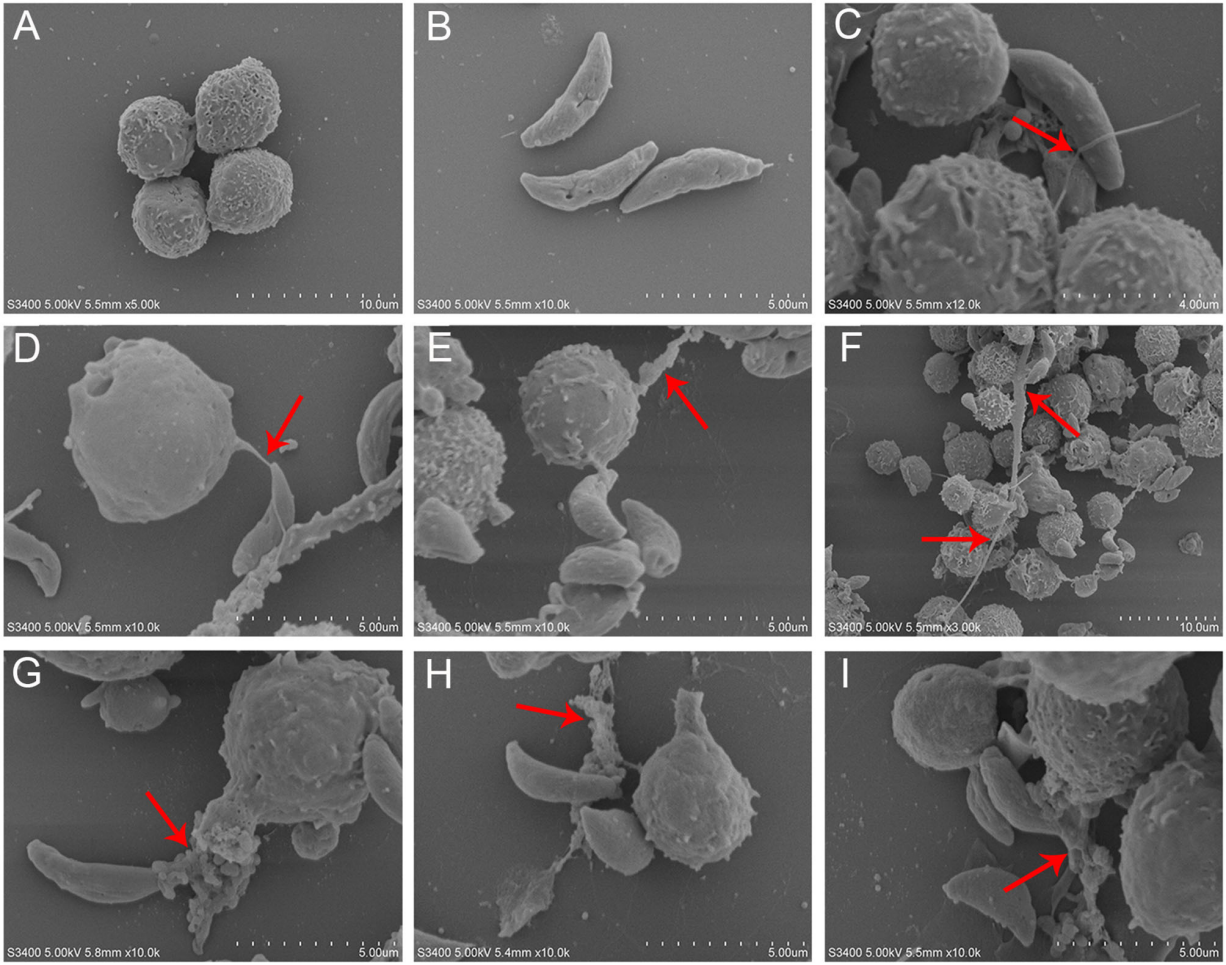
SEM analysis of *T. gondii* tachyzoites-triggered dog NETs release. **(A)** PMNs. **(B)** *T. gondii* tachyzoites. **(C–E)** Thinner NETs-like structures triggered by *T. gondii* tachyzoites. **(F–I)** thicker NETs-like structures triggered by *T. gondii* tachyzoites. Red arrows showed NETs-like structures triggered by *T. gondii* tachyzoites.

*T. gondii* tachyzoites significantly triggered NETs-like structures (Figure 1), and it was found that *T. gondii* tachyzoites were captured by thicker (Figures 1F–I) or thinner (Figures 1C–E) NETs-like structures.

### NE and MPO Co-located With DNA in *T. gondii* Tachyzoites-Triggered NETs Release

**Figure 2:**
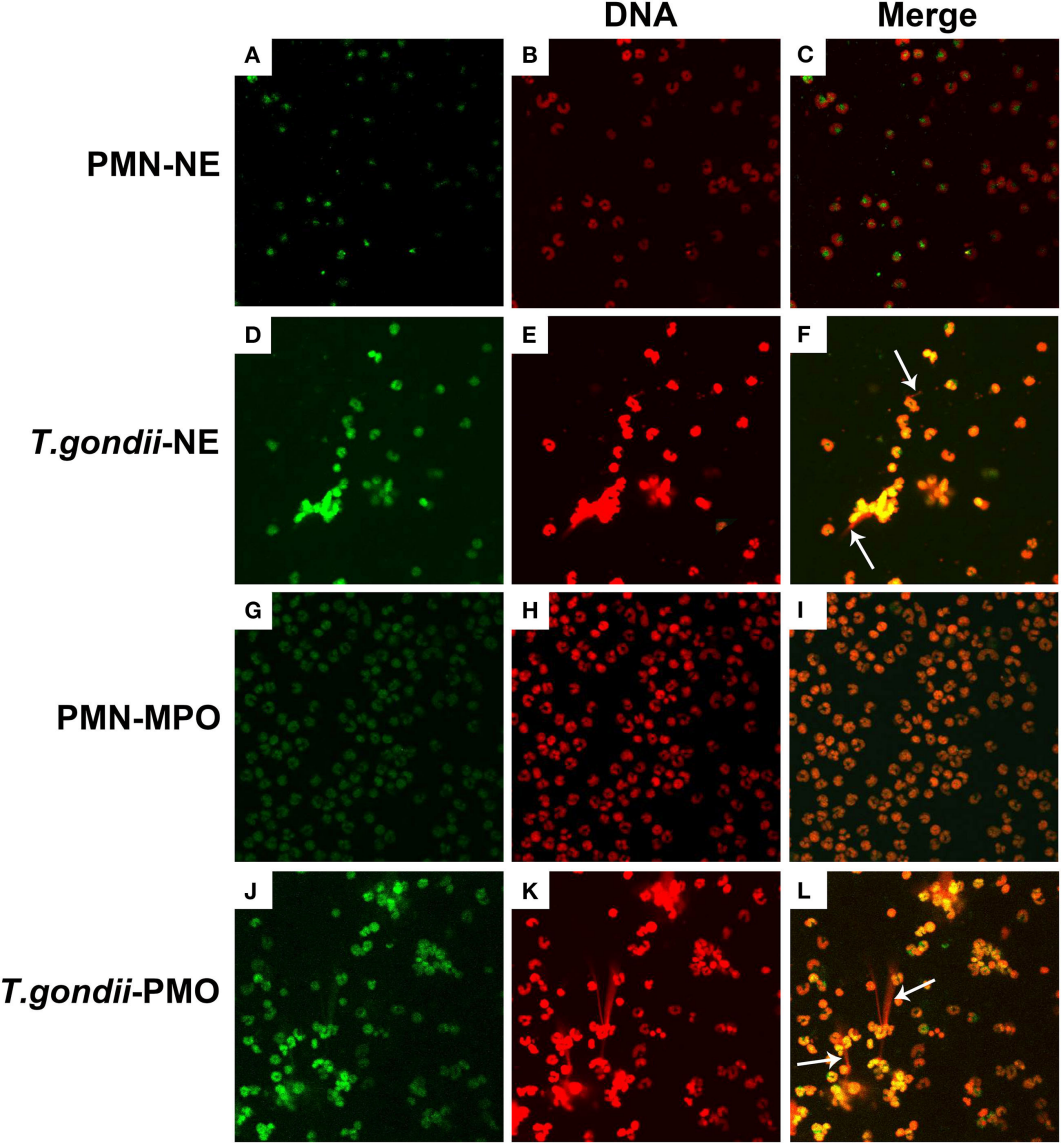
Fluorescence confocal microscopy analysis of *T. gondii* tachyzoites-triggered NETs consisting of NE and MPO with DNA. PMNs were incubated with viable *T. gondii* tachyzoites at a ratio of 1:1 for 90 min. The images were observed and collected by fluorescence confocal microscopy. **(A,D)** NE (Green). **(G,J)** MPO (Green). **(B,E,H,K)** DNA (Red). **(C)** Merge of **(A,B)**. **(F)** Merge of **(D,E)**. **(I)** Merge of **(G,H)**. **(L)** Merge of **(J,K)**. White arrow showed NETs triggered by *T. gondii* tachyzoites.

To confirm, if *T. gondii* tachyzoites-triggered NETs have similar characteristics to the typical NETs-structures, the constituent proteins in *T. gondii* tachyzoites-triggered NETs-like network structures were analyzed. These results showed that *T. gondii* tachyzoites significantly triggered NETs-like structures, and these structures consisted of DNA (Figures 2E,K) decorated with NE (Figure 2D) and MPO (Figure 2J).

### Quantitation of NETs Release Triggered by *T. gondii* Tachyzoites

**Figure 3:**
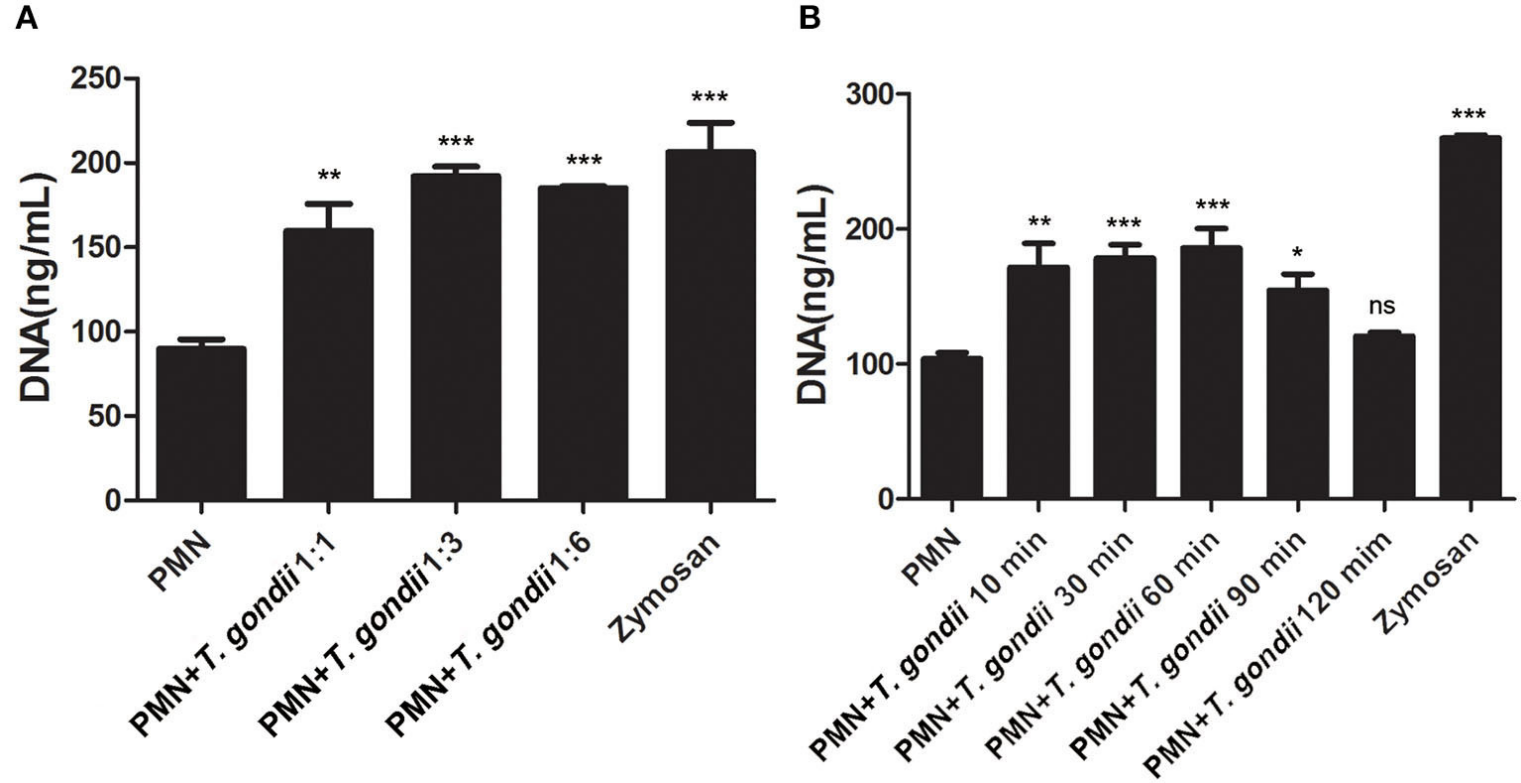
**(A)** *T. gondii*-triggered NETs in a dose-dependent manner. PMNs were incubated with viable *T. gondii* tachyzoites (ratio 1:1, 1:3, or 1:6) for 90 min. Zymosan was used as positive control. Data were expressed as mean ± SD (*n* = 3). **(B)** Dynamic analysis of *T. gondii*-triggered NETs release. PMNs were incubated with viable *T. gondii* tachyzoites (ratio 1:1) for 10, 30, 60, 90, or 120 min. The NETs release triggered by *T. gondii* was quantified with Pico Green and the fluorometric reader. Data were expressed as mean ± SD (*n* = 5). *P* < 0.05 were considered significant (*P < 0.05, **P < 0.01, ***P < 0.001 and “ns,” not significant).

**Figure 4:**
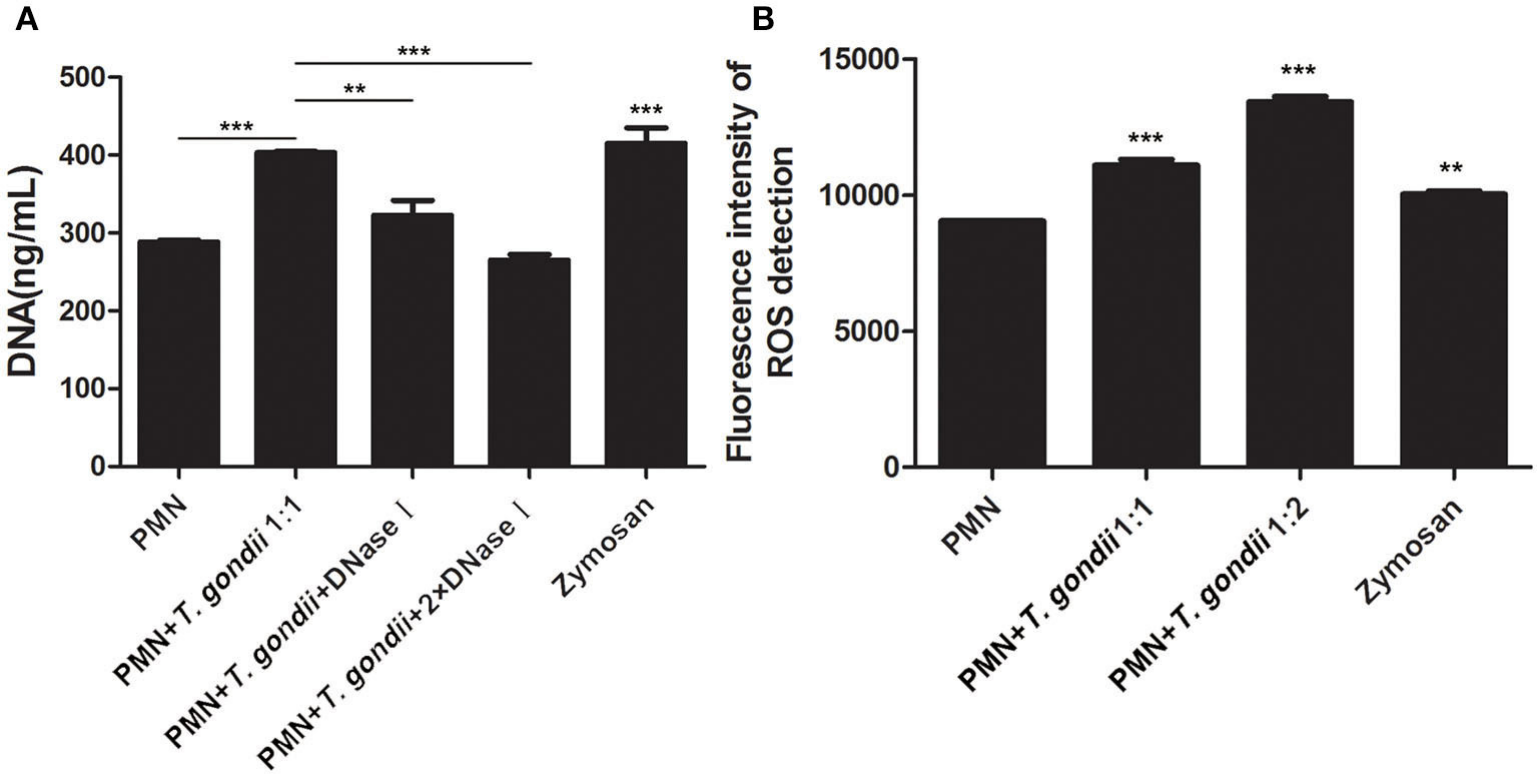
**(A)** *T. gondii*-triggered NETs release degraded by DNase I. PMNs were pretreated with DNase I (90 U) or DNase I (180 U) for 15 min, and then incubated with viable *T. gondii* tachyzoites (ratio 1:1) for 90 min. NETs release triggered by *T. gondii* was quantified with Pico Green and the fluorometric reader. Data were expressed as mean ± SD (*n* = 3). **(B)** Increased ROS levels in *T. gondii* tachyzoites-triggered NETs release. PMNs were incubated with viable *T. gondii* tachyzoites (ratio 1:1 or 1:2) for 90 min. NETs release triggered by *T. gondii* was tested by the fluorometric reader. Data were expressed as mean ± SD (*n* = 3). *P* < 0.05 were considered significant (**P < 0.01 and ***P < 0.001).

As shown in Figure 3A, *T. gondii* tachyzoites significantly triggered NETs release compared to control groups, and the release of NETs was in a dose-dependent manner (*P* < 0.01, *n* = 3). Further experiments revealed that *T. gondii* tachyzoites-triggered NETs release within 60 min which was in a time-independent manner (*P* = 0.002, *n* = 5), and it was of great interest that the increasing of NETs release significantly decreased at the time point of 90 min (*P* = 0.034, *n* = 5) and 120 min (*P* = 0.925, *n* = 5; Figure 3B). It was also shown that DNase I significantly decreased *T. gondii* tachyzoites-triggered NETs release (*P* < 0.01, *n* = 3; Figure 4A), which further confirmed the peculiarity of DNA in these *T. gondii* tachyzoites-triggered NETs structures.

### Increased ROS Levels in *T. gondii* Tachyzoites-Triggered NETs Release

It is reported that NETs formation has been associated with ROS production, thus we analyzed ROS levels in the process of *T. gondii* tachyzoites-triggered NETs release. The results showed that *T. gondii* tachyzoites significantly increased the levels of ROS compared to control groups (*P* < 0.001, *n* = 3; Figure 4B). Furthermore, zymosan also significantly induced ROS production in PMNs (*P* = 0.009, *n* = 3), but lower than that of *T. gondii* tachyzoites (Figure 4B).

### *T. gondii* Tachyzoites-Triggered NETs Release Decreased by NADPH Oxidase-, Rac-, and p38 MAPK Signaling Pathways-Inhibitor Treatments

**Figure 5:**
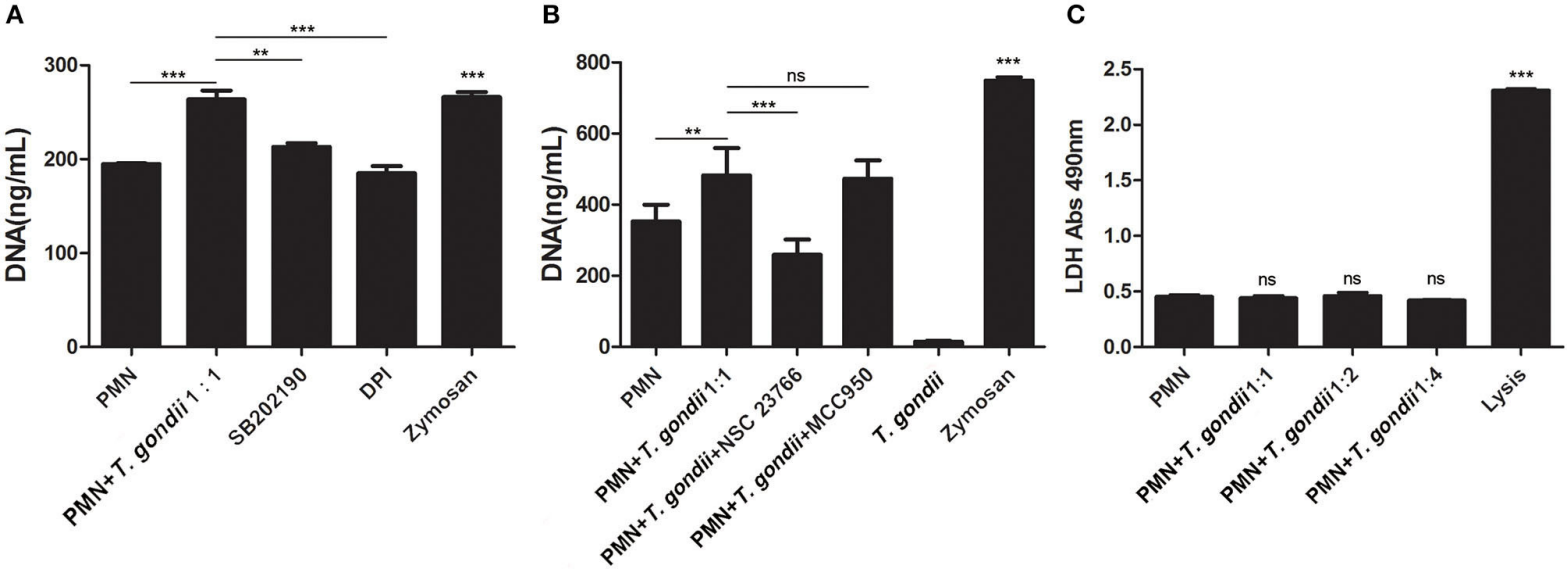
**(A)** *T. gondii*-triggered NETs release inhibited by DPI or SB202190. PMNs were pre-treated with the NADPH oxidase inhibitor DPI or the p38 MAPK signaling pathway inhibitor SB202190 for 15 min, and then incubated with viable *T. gondii* tachyzoites (ratio 1:1) for 90 min. NETs release triggered by *T. gondii* was quantified with Pico Green and the fluorometric reader. Data were expressed as mean ± SD (*n* = 3). **(B)** *T. gondii*-triggered NETs release inhibited by NSC23766. PMNs were pre-treated with the Rac1 activation inhibitor NSC23766 or the NLRP3 inhibitor MCC950 for 15 min, and then incubated with viable *T. gondii* tachyzoites (ratio 1:1) for 90 min. NETs release triggered by *T. gondii* was quantified with Pico Green and the fluorometric reader. Data were expressed as mean ± SD (*n* = 5). *P* < 0.05 were considered significant. **(C)**. No changes of LDH levels in *T. gondii* tachyzoites-triggered NETs release. PMNs were incubated with *T. gondii* tachyzoites (ratio 1:1, 1:2, or 1:4) for 90 min, and LDH activities in supernatant were tested by the LDH kits. The lysis reagent was provided by the kit which was used as positive control. Data were expressed as mean ± SD (*n* = 5). *P* < 0.05 were considered significant (**P < 0.01, ***P < 0.001, and ns, means not significant).

Inhibition experiments revealed that the NADPH oxidase inhibitor DPI (*P* < 0.001), the p38 MAPK signaling pathway inhibitor SB202190 (*P* < 0.01), and the Rac1 activation inhibitor NSC23766 (*P* < 0.001, *n* = 5) significantly decreased the release of *T. gondii* tachyzoites-triggered NETs in varying degrees (Figures 5A,B), while the NLRP3 inhibitor MCC950 slightly decreased NETs release but was not significant (*P* = 0.999, *n* = 5; Figure 5B).

### No Changes of LDH Levels in *T. gondii* Tachyzoites-Triggered NETs Release

LDH is a typical indicator of cell necrosis. To further investigate if cell necrosis also exists in the process of *T. gondii* tachyzoites-triggered NETs release, LDH levels in culture supernatant were examined. As shown in Figure 5C, there was no significant changes in LDH levels were detected in the process of *T. gondii* tachyzoites-triggered NETs release (*P* > 0.05, *n* = 3), but LDH levels were significantly increased by the lysis (provided by LDH kits; *P* < 0.001, *n* = 3).

### *T. gondii* Tachyzoites Escaped From NETs Release

**Figure 6:**
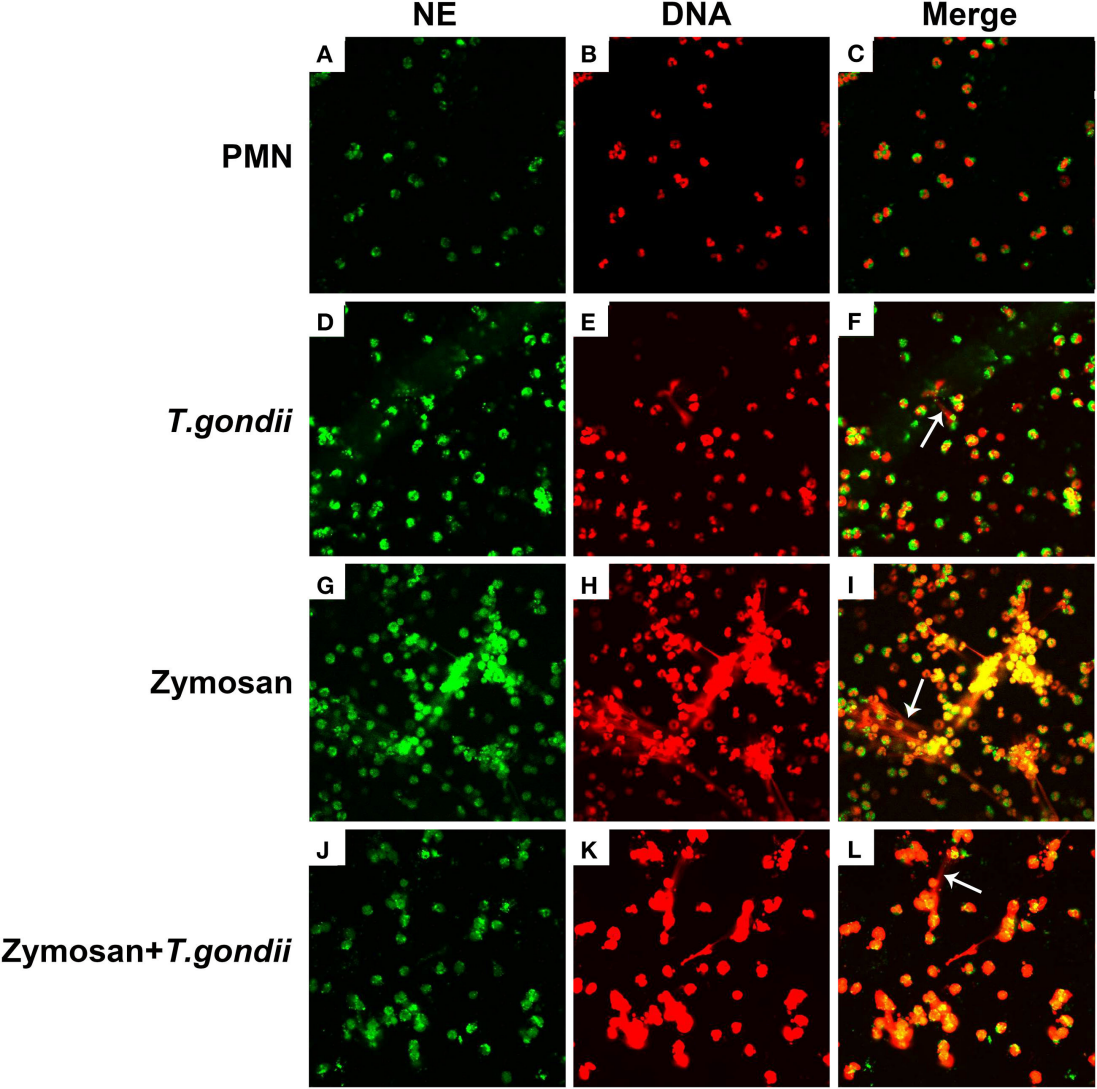
Zymosan-induced NETs (DNA decorated with NE) were degraded by *T. gondii* tachyzoites. PMNs were incubated with viable *T. gondii* tachyzoites (ratio 1:1) or zymosan (1 mg/mL) for 120 min. The images were observed and collected by fluorescence confocal microscopy. **(A,D,G,J)** NE (Green). **(B,E,H,K)** DNA (Red). **(C)** Merge of **(A,B)**. **(F)** Merge of **(D,E)**. **(I)** Merge of **(G,H)**. **(L)** Merge of **(J,K)**. White arrow showed NETs triggered by *T. gondii* tachyzoites.

**Figure 7:**
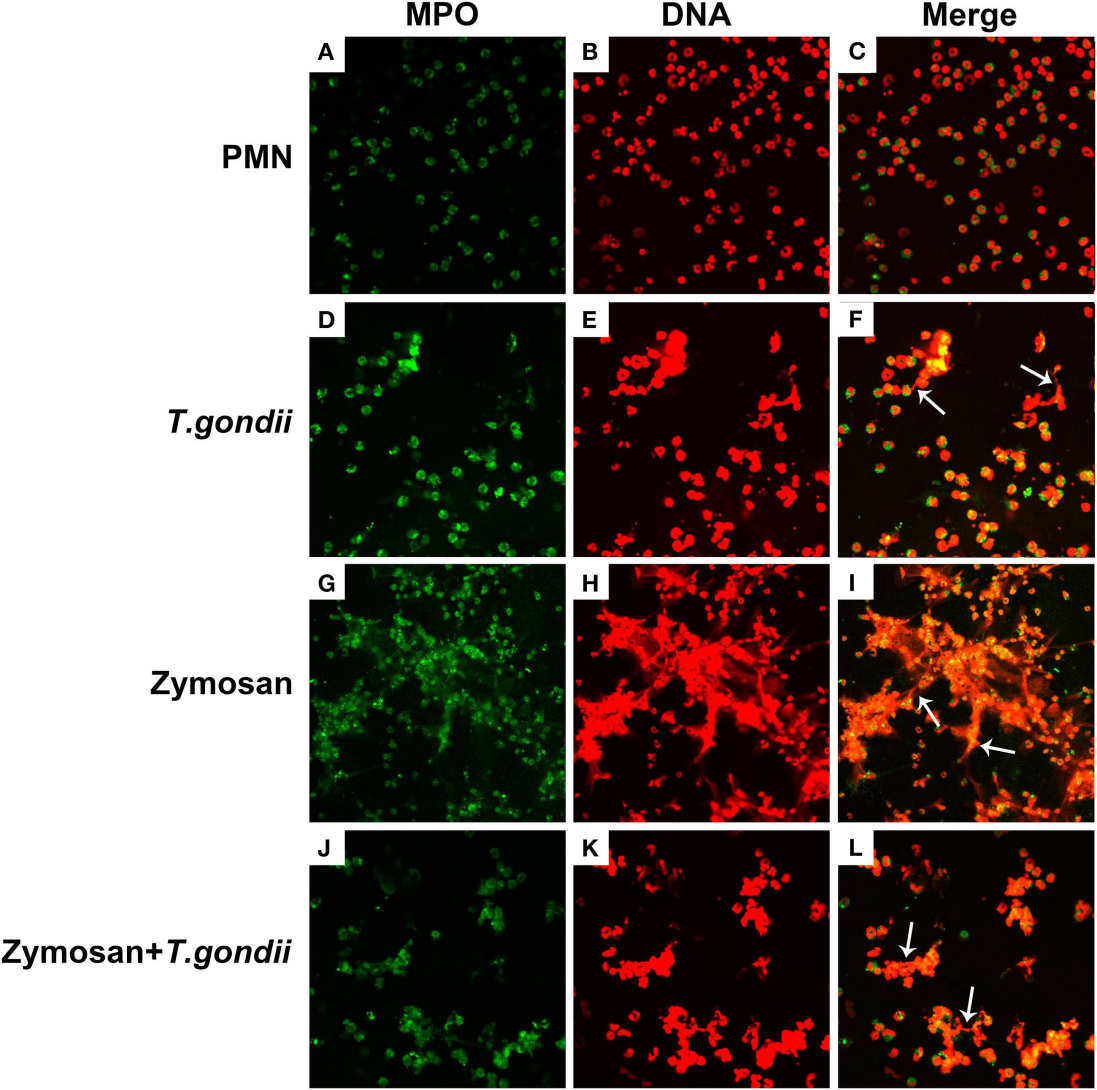
Zymosan-induced NETs (DNA decorated with MPO) were degraded by *T. gondii* tachyzoites. PMNs were incubated with viable *T. gondii* tachyzoites (ratio 1:1) or zymosan (1 mg/mL) for 120 min. The images were observed and collected by fluorescence confocal microscopy. **(A,D,G,J)** MPO (Green). **(B,E,H,K)** DNA (Red). **(C)** Merge of **(A,B)**. **(F)** Merge of **(D,E)**. **(I)** Merge of **(G,H)**. **(L)** Merge of **(J,K)**. White arrow showed NETs triggered by *T. gondii* tachyzoites.

PMNs were co-treated with zymosan and *T. gondii* tachyzoites to clarify if *T. gondii* could escape from NETs. As shown in Figures 6, 7, NETs release triggered by *T. gondii* tachyzoites was slightly observed at the time point of 120 min, while zymosan significantly triggered NETs compared to negative controls. However, the release of zymosan-triggered NETs was strikingly degraded by *T. gondii* tachyzoites treatment (Figures 6L, 7L). These NETs structures triggered by *T. gondii* tachyzoites consisted of NE (Figures 6D,G,J) and MPO (Figures 7D,G,J) with DNA (Figures 6E,H,K, 7E,H,K).

## Discussion

In the present study, NETs release triggered by *T. gondii* tachyzoites has been demonstrated in dogs for the first time, just like that in humans, sheep, or harbor seals (Abi Abdallah et al., 2012; Reichel et al., 2015; Yildiz et al., 2017). It was clear that *T. gondii* tachyzoites were captured by NETs-like structures (Figure 1), and these structures consisted of DNA decorated with NE and MPO (Figure 2), which confirmed that similar and typical features of NETs-like structures were observed in other parasites such as: *Neospora caninum* (Wei et al., 2016), *Eimeria bovis* (Munoz-Caro et al., 2015a), *Cryptosporidium parvum* (Munoz-Caro et al., 2015b), and *Eimeria arloingi* (Silva et al., 2014). Quantitation analysis demonstrated *T. gondii* tachyzoites triggered NETs release was in a dose-dependent manner. These results have been suggested that one part of *T. gondii* tachyzoites would be captured and then killed by NETs in dogs. A further quantitative experiment also revealed that *T. gondii* tachyzoites-triggered NETs release was in a time-independent manner, and it was of great interest that the increase in NETs release was significantly decreased at the time point of 120 min. In addition, the release of NETs triggered *T. gondii* tachyzoites was also degraded by DNase I (Figure 4A). All these results suggest that another part of *T. gondii* tachyzoites could also evade capture by these NETs after 90 min.

Given that ROS, NADPH oxidase, Rac, or p38 signaling pathways are involved in the mechanism of several pathogen-induced NETs releases (Lim et al., 2011; Munoz-Caro et al., 2015a,b; Wei et al., 2016, 2018a; Gavillet et al., 2018), and NLRP3 inflammasomes can be activated during *T. gondii* infection (Gorfu et al., 2014; Quan et al., 2018), we investigated if these molecular or signaling pathways also participate in *T. gondii* tachyzoites-triggered NETs formation. It was found that inhibitors of the NADPH oxidase, Rac 1, or p38 MAPK signaling pathways but not the inhibitor of NLRP3 significantly inhibited NETs release (Figures 5A,B), which suggested that NADPH oxidase, Rac 1, and p38 MAPK signaling pathways were relevant to *T. gondii* tachyzoites-triggered NETs. Further results showed that *T. gondii* tachyzoites significantly increased ROS levels but not LDH levels in the process of NETs release, proving that ROS was also related to *T. gondii* tachyzoites-triggered NETs. Zymosan used as the positive stimuli could also significantly induce ROS production in NETs release as shown in previous research (Wei et al., 2016, 2018a,b), but lower than that of *T. gondii* tachyzoites. We deduced that there are possibly two main reasons for the results. The first one is that the type of stimuli are different for inducing NETs release. NETs release is a ROS -dependent or -independent process, these two ways may both exist in NETs release. The other reason is that the amount of the same stimuli significantly affects ROS production in the process of NETs release. Taken together, the mechanism of *T. gondii* tachyzoites-triggered NETs release was a ROS-, NADPH oxidase-, Rac 1-, or p38 MAPK- signaling pathway dependent process. In this aspect, no significant species-difference of *T. gondii* tachyzoites-triggered NETs were found between harbor seals and dogs.

On the basis of the quantitative results that NETs release triggered by *T. gondii* tachyzoites was markedly decreased at the time point of 120 min, we speculated that *T. gondii* tachyzoites could degrade NETs structures and escape from the NETs-based capture strategy. To provide further evidence, PMNs were co-treated with zymosan and *T. gondii* tachyzoites for fluorescence confocal microscopy analysis. Interestingly, zymosan-triggered NETs release was strikingly degraded by *T. gondii* tachyzoites treatment that confirmed our suspicions. In other words, there must be one or more DNase-like enzyme existing in *T. gondii* to degrade NETs structures. However, predicting and identifying these DNase-like enzymes in *T. gondii* still needs further research, which will help understand the biological functions and mechanisms of *T. gondii* escape from the NETs-based capture strategy.

In conclusion, the NETs-based effector mechanism worked in dogs during *T. gondii* infection, and the regulatory mechanism undergoing *T. gondii*-triggered NETs release was associated with ROS-, NADPH oxidase-, Rac 1-, or p38 MAPK- signaling pathways. Moreover, it was found that *T. gondii* escaped from the NETs-based capture strategy, but the escape mechanism still needs careful study.

## Data Availability Statement

All datasets generated for this study are included in the article/supplementary material.

## Ethics Statement

The animal study was reviewed and approved by Animal experiments were approved by the Ethics Committee on the Care and Use of Laboratory Animals of Foshan University. Written informed consent was obtained from the owners for the participation of their animals in this study.

## Author Contributions

ZY and QL conceived and designed the project and experiments. ZWe, ZWa, XL, CW, ZH, DW, and YZ carried out the experiments. ZWe and XZ performed the statistical analysis. ZWa wrote the manuscript. QL revised the manuscript. All authors reviewed the manuscript.

## Conflict of Interest

The authors declare that the research was conducted in the absence of any commercial or financial relationships that could be construed as a potential conflict of interest.

## Abbreviations

NET: neutrophils extracellular trap
SEM: scanning electron microscopy
PMN: polymorphonuclear neutrophil
NE: neutrophil elastase
MPO: myeloperoxidase
ROS: reactive oxygen species
MAPK: mitogen-activated protein kinase
LDH: lactate dehydrogenase
SOCE: store-operated calcium entry
DPI: Diphenyleneiodonium chloride.
